# A Preclinical Medical School Curriculum on Firearm Violence to Develop Patient Counseling and Foundational Health Policy Skills

**DOI:** 10.1080/10872981.2021.1984177

**Published:** 2021-10-21

**Authors:** Natalie Kucirek, Christine Studenmund, Daniella M. Cordero, Megha Garg

**Affiliations:** a Medical Student, University of California San Francisco School of Medicine, San Francisco, CA, USA; b Resident Physician, Division of Plastic and Reconstructive Surgery, University of Washington, Seattle, WA, USA; c Associate, Department of Medicine, San Francisco VA Medical Center and University of California San Francisco, San Francisco, CA, USA

**Keywords:** Gun violence, undergraduate medical education, health communication, public health, firearm safety, curriculum development

## Abstract

**Background:**

Firearm violence is a unique public health crisis in the USA (US). A majority of U.S. physicians believe they should discuss firearm safety with patients. However, little education on firearm injury prevention and counseling exists in medical school. We sought to address this gap by creating a curriculum on firearm violence as a part of a required preclinical medical school course focused on health policy issues.

**Methods:**

The Kerns 6-step model for curriculum development was used to define the problem and assess learner needs. The two-hour small group session was co-authored by a student and faculty member to address the course theme of health policy as applied to firearm violence. The Issue-Attention Cycle, history of firearm policy, and US politics were incorporated from published literature, with a patient counseling role-play added in 2019.

**Results:**

The ‘Current Case in Health Policy – Firearm Violence’ small group was implemented in 2018 and 2019 for all first-year medical students. Of the 2018 student evaluations, 57% selected this small group as the most valuable in the course. In a follow-up survey in 2020, 78% of the respondents agreed that they felt more confident counseling patients on firearm safety following the role-play.

**Conclusion:**

Students broadly endorsed the incorporation of firearm policy and counseling skills into medical education. This curriculum can be adapted for learners at all stages of training, especially given the limited exposure to this topic in medical education.

## Introduction

In 2018, the shooting at Marjory Stoneman Douglas High School in Parkland, Florida, amplified existing discourse around firearm violence as a public health issue in the USA (US). In 2017, 39,773 people died from firearm-related injuries in the US, surpassing the number of deaths from motor-vehicle crashes (38,659) and alcohol (35,823) [1].

Studies across specialties have found that a majority of physicians believe they should be involved in reducing firearm injury, but few actually perform such interventions [2–4]. These results were echoed in a study of students at 16 medical schools, in which 84% of those surveyed indicated that it was at least somewhat important to discuss firearm storage and access with patients, but only 4% usually or always did so [5].

Despite demands to address firearm violence as a public health crisis by numerous professional organizations [6,7], medical education on firearms remains sparse. A 2016 systematic review of firearm safety education for medical trainees and providers found only four studies of such programs, exposing a need for further curricular development [8].

We created a curriculum for first-year medical students to address the public health crisis of firearm violence, while also teaching students how domestic policies and legislation influence the practice of medicine. The curriculum included space for students to discuss the personal impact of firearms and related legislation, and to practice patient counseling.

## Materials and methods

In March 2018, we developed a curriculum incorporating the epidemic of firearm violence into Health and Society (H&S), a three-week required preclinical course for all first-year medical students at the University of California, San Francisco (UCSF). We used Kern’s 6-Step Method for medical curricular development to identify educational needs, objectives, and learning format [9]. The components of the Kern’s process were informed by previous student course evaluations for H&S, core competencies for medical students at our institution [10], and literature review of current curricula on firearm violence.

A first-year medical student with an interest in firearm violence prevention and a physician H&S course director co-authored a one-time, two-hour small group session (Appendix A). Students participated in groups of 10–15 with clinical/social sciences faculty or medical student teaching assistants as facilitators. Facilitators received an orientation that included content review, training on navigating difficult conversations that may arise in the context of the small group, as well as a Facilitator Guide (Appendix B) with key points and guidance on time per section to standardize implementation of the curriculum.

Learners completed required pre-readings on public health theory, epidemiological research, legislation history, and research compilations [11–14]. Optional pre-work included podcasts, videos, and first-person accounts [15–17]. Students also completed the American Medical Association’s online module ‘The Physician’s Role in Promoting Firearm Safety.’[18]

The small group had 4 sections: 1) Reflection on the role of firearms in students’ communities and personal lives, 2) Discussion of the Issue-Attention Cycle, a framework that describes the public’s response to societal issues with a series of five predictable stages [11], and its application to public health crises 3) Patient counseling role-play on firearm safety, and 4) Discussion of firearm regulation policy and legislation in the US. The small group student and facilitator guides are included as Appendices A and B.

Feedback on the initial curriculum in 2018 and the updated curriculum in 2019 was elicited through student evaluations of the entire H&S course. It is our institution’s practice to sample a subset of students for post-course evaluation to mitigate survey request fatigue. Accordingly, 49 students in 2018 and 51 students in 2019 were randomly assigned an H&S course evaluation within 2 weeks of completing the course, we also developed an optional, anonymous survey using Qualtrics survey software (Qualtrics; Provo, UT) administered in June 2020 as a one-year follow-up for the 2019 edition of the small group to assess student perceptions of the value of the curriculum and confidence in knowledge from learning objectives. The survey included Likert scale questions based on the learning objectives and a free response section for further comments (Appendix C). This survey was distributed to all of the students who participated in the 2019 course. Quantitative questions were analyzed using descriptive statistics.

## Results

This curriculum was implemented in the 2018 H&S course for 151 students. The overall H&S course evaluation, covering all the content in the course, was distributed to 49 students and was completed by 47 students (96%). Of the respondents, 57% (n = 27) selected this small group as the most valuable of the sessions in the course. In qualitative comments, students indicated a desire to further practice firearm safety discussions with patients. This feedback led to the addition of the role-play activity in the current curriculum.

The updated curriculum was administered to 157 first-year medical students in 2019. The overall H&S course evaluation for 2019 was distributed to 51 students and was completed by 47 students (92%). In qualitative comments, students indicated that the small group was valuable and applicable to current events and that a background lecture given before the small group would be a helpful addition to the curriculum.

Of the 157 students from the 2019 curriculum who received the optional one-year follow-up survey in June 2020, 36 students responded (23%). The majority of students ‘strongly agreed’ or ‘agreed’ on the importance of physicians understanding the impact of firearm violence (92%) and indicated increased confidence in the ability to counsel patients on firearm safety (78%). The results are summarized in Table 1.

**Table 1.** Survey of students 1 year after participation in the 2019 firearm curriculum (n = 36)

Statement	Response % (n)					Mean
	1	2	3	4	5	
I believe it is important for physicians to understand the impact that firearm violence and related health policies can have on patients.	3% (1)	3% (1)	3% (1)	25% (9)	67% (24)	4.5
I understand the concept of the Issue-Attention Cycle and the implications that it has for solving a health policy issue, such as firearm regulation.	3% (1)	3% (1)	6% (2)	33% (12)	56% (20)	4.4
The ‘Current Case in Health Policy – Firearm Violence’ small group allowed me time and space to reflect on the impact that a health policy, specifically firearm regulation, has had on my own experiences.	6% (2)	3% (1)	14% (5)	56% (20)	22% (8)	3.9
I felt more confident in my ability to counsel a patient/family about firearm safety after practicing during a role play activity during this small group.	3% (1)	6% (2)	14% (5)	72% (26)	6% (2)	3.7
This small group influenced the way I think about firearm violence and related health policies and laws, including their potential impact on my patients.	6% (2)	11% (4)	11% (4)	56% (20)	17% (6)	3.7

***Key***: 1 = strongly disagree, 2 = disagree, 3 = neutral, 4 = agree, 5 = strongly agree

In free response comments from the 2020 survey, students stressed the importance of firearm safety in medical education. Students were also interested in applying the Issue-Attention Cycle policy framework to other public health crises, including police violence. Some also proposed improvements, including facilitation by content experts and education on harm reduction techniques.

## Discussion

We created a small group curriculum for medical students that integrates a health policy framework with firearm access screening and counseling practice. We posit that starting conversations around firearms within peer groups helps to build comfort in discussing this potentially challenging issue with patients. We also argue that providing background on the policy framework and societal perceptions surrounding firearms better prepares students for injury prevention counseling. In response to student feedback highlighting the importance of translating didactic knowledge into practice, we modified the session to include a patient counseling role play in 2019.

We developed this curriculum for first-year, preclinical medical students. However, this small group could be easily incorporated as a didactic for clinical students on clerkships such as pediatrics or psychiatry, or used to serve more advanced resident trainees and practicing physicians, as these are relatively new topics in medical education. This curriculum could also be extended to include interprofessional health-care providers with modification of the role-play element.

The survey responses indicate that students believe it is important for physicians to understand the impact of firearm violence on their patients. This matches findings in the existing literature that most physicians view firearm violence as a public health issue [3,4,6,7]. Seventy-eight percent of respondents either agreed that the small group improved their confidence in firearm safety counseling. In the future, incorporating a session in which students practice their firearm counseling skills with standardized patients could further improve student confidence.

For the 2020 survey results, several limitations must be considered. We administered the evaluation survey over 1 year after the session. While this may influence the results due to recall bias, it provides insight into confidence about their knowledge from the curriculum. The 23% response rate limits the generalizability due to possible nonresponse bias. However, we believe that these findings can still provide valuable guidance for future curricular development, particularly given the limited published research on this topic to date.

Several comments in the survey responses expressed a desire to select content experts as small group facilitators. This may be challenging at institutions that do not have physicians or community members who specialize in firearm violence prevention. While content expertise is always valuable, we focused on the skill of facilitating challenging conversations that is applicable for educators across a variety of topics. Students also suggested adding a general lecture to the course regarding firearm violence prior to the small group, which may be a more feasible addition to the content across institutions.

Another potential challenge is the perception that discussing firearm safety is political or inappropriate for the medical school curriculum. We emphasize that reducing firearm injury is broadly supported by non-partisan physician organizations across specialties [2,4,7] and should be approached as a public health intervention. We are also fortunate that our institutional leadership supported our efforts to include these topics in the medical school curriculum and hope that our example will provide support to others who may face challenges from their administration. Furthermore, the general public appears open to physician counseling on firearms, as 66% of surveyed US adult participants in one study believed it to be ‘at least sometimes appropriate.’ [19] Future studies should explore these topics brought up in our survey comments to inform pedagogy and student response to controversial topics in medical education.

We also recognize that students with minority opinions may feel reticent to express themselves. We emphasize the importance of clear expectation-setting at the start and inviting a breadth of opinions since the patients we serve will have similarly diverse beliefs, supported by the above-noted study in which one-third of US adults did not think discussing firearms with a physician was appropriate [19].A larger group debrief could also be considered in subsequent versions of the curriculum.

We envision that the policy framework of this curriculum, the Issue-Attention Cycle, could be adapted to other public health issues like the impact of climate change, systemic racism, or police violence. Future iterations of the curriculum should also discuss the effects of the 2020 US Congressional appropriation bill, which granted $25 million to the National Institutes of Health (NIH) and the Centers for Disease Control and Prevention (CDC) to study firearm injury prevention and represented the first federal funding of firearm research since 1996, during the final section of the small group [20].

## Appendix A. Current Case in Health Policy – Firearm Violence

**Session Type: Small Group**

**Student Guide 2019**

**Objectives**

- Describe the Issue-Attention Cycle and its implications for solving a health policy issue such as firearm regulation
- Understand the history of firearm violence/firearm regulation in the USA and the politics of policy change
- Reflect on the impact of firearm regulation policies on personal experiences
- Practice counseling a patient on firearm safety

**Key Words**

- Issue-attention cycle, window of opportunity, firearm violence, risk assessment, patient counseling

**Session Timeline**

Intro and review group norms (5 minutes)

Part I: Reflection (20–30 minutes)

Part II: Issue-Attention Cycle of Domestic Policy (20 minutes)

Part III: Counseling patients on firearm safety (20–30 minutes)

Part IV: Firearm regulation history (20 minutes)

Wrap up (5 minutes)

**Part I: Reflection (20–30 minutes)**

Before starting, please review community norms set by your class for your small groups.

Discuss with your group the following questions related to firearms, firearm regulation, and firearm violence. You may pair share or discuss in the larger group. Please only share what you feel comfortable sharing.

- What has your personal experience with firearms been? Do you, family members, or anyone close to you own or have access to firearms?
- What is your stance on firearm regulation, including legislation and policies that aim to reduce firearm injury and that impact aspects of firearm access and ownership, in the US?
- Have you observed or led a conversation in a medical setting with patients about firearm safety or firearm ownership/access?
- How has firearm violence in the US impacted you? What, if any, role do you think you will have in the firearm violence debate in the US?

**Part II: Issue-Attention Cycle of Domestic Policy (20 minutes)**

Issue-attention cycle:

- Review the main concepts of the issue-attention cycle (5 minutes)
- In what stage of the issue-attention cycle would you argue that firearm regulation/firearm violence is in now? Where has it been in the past year? In your lifetime?
- Kingdon’s policy streams model (in *Agendas, Alternatives, and Public Policies*, 2011) proposes a ‘window of opportunity’ in which three forces align for policy change: 1) a problem gets recognized and defined, 2) a solution is available, and 3) there is political will/political climate open for change.
  - Do you think there is a window of opportunity right now to change firearm regulation, including legislation and policies that aim to reduce firearm injury and that impact aspects of firearm access and ownership? Why or why not?
- Can you as a health-care provider disrupt or contribute to this cycle? What is the responsibility of a medical provider to engage in this debate?
- Reflect on the effective response of the students in Parkland, Florida, in 2018 in the firearm regulation conversation and the larger response from students around the country (reference the giffords.org article from your readings). What makes this group of students different from others who have been impacted by firearm violence? Is there something special about this generation compared to others (i.e., generational ‘spirit’, social media, etc.).

**Part III: Counseling patients on firearm safety – Role-Play (20–30 minutes)**

This is an opportunity to practice some of the skills presented in the AMA CME session ‘The Physician’s Role in Promoting Firearm Safety’.

Please pair with a partner: one of you will assume the role of the pediatrician and the other ofa parent in a well-child visit. Practice using the language you have learnt in risk assessment and counseling around firearms. Please reference the AMA information sheets included in the small group guide for this activity.

Provider Information:

CC/ID: Jessica is a 5-year-old girl presenting to for a well-child exam to her pediatrician’s office

HPI: The patient is meeting all developmental milestones and up to date on vaccines Social History: She lives with her mother and grandfather

How would you:

- Ask about firearms in the home, and how accessible they are?
- Provide counseling on firearm safety?

Parent/Caretaker Information:

- You own a single firearm that is stored in your closet. It is loaded and stored in an unlocked box on the top shelf.
- When asked if Jessica could access it, you say ‘Oh, no I don’t think she would do that. Plus, it is too high up. Although I guess she could use a chair to climb up there. Sometimes she goes in there to play dress up.’
- You are concerned about the cost of gun safety.

**Part IV: Firearm regulation history (20–30 minutes)**

Discuss the following:

- Why has there been no CDC-funded research on firearm violence in over 20 years?
- Given the current situation, what further studies need to be done on this issue? What creative ways can we think of to carry these out or fund them?

**Wrap Up (5 minutes)**

Please spend a few minutes sharing what you learned from this session, and any questions you have remaining about this issue.

**Appendices were reviewed and updated to include the most current, unbiased terminology available at the time of publication.*

## Appendix B. Current Case in Health Policy – Firearm Violence

**Session Type: Small Group**

**Facilitator Guide 2019**

**Objectives**

- Describe the Issue-Attention Cycle and its implications for solving a health policy issue such as firearm regulation
- Understand the history of firearm violence/firearm regulation in the USA and the politics of policy change
- Reflect on the impact of firearm regulation policies on personal experiences
- Practice counseling a patient on firearm safety

**Key Words**

- Issue-attention cycle, window of opportunity, firearm violence, risk assessment, patient counseling

**Session Timeline**

Intro and review group norms (5 minutes)

Part I: Reflection (20–30 minutes)

Part II: Issue-Attention Cycle of Domestic Policy (20 minutes)

Part III: Counseling patients on firearm safety (20–30 minutes)

Part IV: Firearm regulation history (20 minutes)

Wrap up (5 minutes)

**Part I: Reflection (20–30 minutes)**

Before starting, please review community norms set by your class for your small groups.

*Facilitator: Please check in with students about whether they would like to review their norms together before starting the small group, or let you know of any small group specific norms that they follow.*

Discuss with your group the following questions related to firearms, firearm regulation, and firearm violence. You may pair share or discuss in the larger group. Please only share what you feel comfortable sharing.

- What has your personal experience with firearms been? Do you, family members, or anyone close to you own or have access to firearms?
- What is your stance on firearm regulation, including legislation and policies that aim to reduce firearm injury and that impact aspects of firearm access and ownership, in the US?
- Have you observed or led a conversation in a medical setting with patients about firearm safety or firearm ownership/access?
- How has firearm violence in the US impacted you? What, if any, role do you think you will have in the firearm violence debate in the US?

*Facilitator: It is expected that students will have different backgrounds, experiences, and opinions about firearms, and this could be an emotional conversation for some. This is where the norms and expectations of the group will come in. As the facilitator, please feel empowered to express your own doubts, confusions, or highlight areas that may need further discussion later. It’s ok to not arrive at any conclusions about these topics, and we encourage you to move on to the policy aspects of this small group and come back to any unfinished topics at the end of the small group or the beginning of your next session if needed.*

**Part II: Issue-Attention Cycle of Domestic Policy (20 minutes)**

Issue-attention cycle:

- Review the main concepts of the issue-attention cycle (5 minutes).

*This discussion of the Issue Attention Cycle is a key takeaway for the small group. Here are some key points from their reading about the cycle:*

*Stages of the Issue Attention Cycle*:

- ***Pre-problem stage****: Highly undesirable social condition exists (i.e., malnutrition, poverty) but has not yet caught public attention.*
- ***Alarmed Discovery and Euphoric Enthusiasm****: As a result of some dramatic series of events, public becomes aware of and alarmed about evils of a particular problem. Public is enthusiastic about the ability to solve the problem.*
- ***Realizing the cost of significant progress****: The cost of solving the problem will be high, not only money but also sacrifices by large groups of the population (i.e., smog regulation might affect people’s ability to drive freely). Sometimes can be mitigated by technological progress.*
- ***Gradual decline of intense public interest****: As people realize how difficult the problem is to solve, they either 1) get discouraged, 2) suppress their discouragement by not thinking about the problem anymore, 3) get bored with the issue. Some other issue is usually entering Stage 2 by now to grab people’s attention.*
- ***The post problem stage****: Issue moves into a period of sporadic public interest, but there may have been some changes that have occurred by now, and the issue will likely always have more prominence and attention than something in the pre-problem stage.*
- In what stage of the issue-attention cycle would you argue that firearm regulation/firearm violence is in now? Where has it been in the past year? In your lifetime?

*No one correct answer here, as long as students can provide a rationale for their answers. Some students may argue that currently firearm regulation/firearm violence is in an extended Alarmed Discovery/Euphoric Enthusiasm stage, as evidenced by the ongoing student activism, physician activism (#ThisIsOurLane movement), and significant policy change since the Parkland shooting (see Giffords Law Center article). However, it would also be reasonable to argue that there is declining momentum and that we are beginning to realize the cost of significant progress and/or that there is now declining public interest. Responses may vary widely between students depending on their relationship to the issue.*

*It is important to note that there has been significant re-emergence of this issue with resultant change over the last year. Previously this issue was likely in the post-problem stage, as every shooting brings some temporary publicity to firearm regulation but without significant change. During a lifetime (which will differ depending on who is in the room), firearm regulation/firearm violence may have gone through all of these stages*.

- Kingdon’s policy streams model (in *Agendas, Alternatives, and Public Policies*, 2011) proposes a ‘window of opportunity’ in which three forces align for policy change: 1) a problem gets recognized and defined, 2) a solution is available, and 3) there is political will/political climate open for change.
  - Do you think there is a window of opportunity right now to change firearm regulation, including legislation and policies that aim to reduce firearm injury and that impact aspects of firearm access and ownership? Why or why not?

*Again, no right or wrong answer here, but fair to argue that right now is a window of opportunity. A couple of years ago, it would have been hard to believe that firearm regulation would be on the table in Congress and that the NRA’s stronghold on politicians could wane. However, also fair to propose the idea that this issue has become less central in the past year and the window is closing. As an example, another issue that had major changes occur during a ‘window of opportunity’ is the passage of the ACA in 2010*.

- Can you as a health-care provider disrupt or contribute to this cycle? What is the responsibility of a medical provider to engage in this debate?

*Students may have strong opinions about this issue. The challenge here will be to engage any dissenting voice in the room. Some ideas for ways that a physician can contribute to the conversation*:

- *Share stories of caring for patients who are victims of firearm violence in public forum (reference NY Times op-ed by a physician from optional pre-reading)*
- *Visit legislators either on behalf of a medical organization or a local group of physicians*
- *Educate your own patients and their families about firearm safety best practices (advocacy for individual patient)*
- *Engage in activism like protests, marches, though would need to determine whether this is in your role as a physician or a private citizen*

- Reflect on the effective response of the students in Parkland, Florida, in 2018 in the firearm regulation conversation and the larger response from students around the country (reference the giffords.org article from your readings). What makes this group of students different from others who have been impacted by firearm violence? Is there something special about this generation compared to others (i.e., generational ‘spirit’, social media, etc.).

*Let the students lead on answering this question. Some ideas include that this group of students was older than the students who were victims of the Sandy Hook Elementary School shooting in 2012. This is also a generation that has grown up with social media and perhaps is able to amplify their voice in a different way. Some may also observe that this group of students received more media coverage due to privilege, while every day firearm violence that disproportionately affects lower SES communities of color does not command the same attention.*

**Part III: Counseling patients on firearm safety – Role-Play (20–30 minutes)**

This is an opportunity to practice some skills presented in the AMA CME session ‘The Physician’s Role in Promoting Firearm Safety’.

Please pair with a partner: one of you will assume the role of the pediatrician and the other of a parent in a well-child visit. Practice using the language you have learnt in risk assessment and counseling around firearms. Please reference the AMA information sheets included in the small group guide for this activity.

Provider Information:

CC/ID: Jessica is a 5-year-old girl presenting to for a well-child exam to her pediatrician’s office

HPI: The patient is meeting all developmental milestones and up to date on vaccines Social History: She lives with her mother and grandfather

How would you:

- Ask about firearms in the home, and how accessible they are?
- Provide counseling on firearm safety?

Parent/Caretaker Information:

- You own a single firearm that is stored in your closet. It is loaded and stored in an unlocked box on the top shelf.
- When asked if Jessica could access it, you say ‘Oh, no I don’t think she would do that. Plus, it is too high up. Although I guess she could use a chair to climb up there. Sometimes she goes in there to play dress up.’
- You are concerned about the cost of gun safety.

*If time allows, switch roles and practice again. If the students are interested, for the second go around feel free to have the parent/caretaker create a different scenario in response to the firearm safety discussion questions from the provider.*

*Here, the most important objective is for students to begin practicing language to gain clarity about the goals (risk reduction!), and confidence in the language used to counsel patients on firearm safety.*

*Please encourage them to remember the group norms set in the beginning of the course, and to interact openly and respectfully.*

*Remember that the goal is to reduce risk, NOT to condemn firearm ownership. Remind students if needed that most firearm owners consider safety a top priority.*

*Students are encouraged to utilize the AMA information sheets attached to this session outlining best practices for counseling patients on firearm safety and provide options for safe gun storage (especially since, in this scenario, the cost of gun safety is a concern for the family). Here is an abbreviated outline of the AMA framework*:

1. *Clarify whether*:
  - *Firearms are stored unloaded*
  - *Firearms are secured with a locking device*
  - *Ammunition is stored separately*
2. *Counsel*
  - *If the firearms are not being stored safely, explore the appropriate actions the*

*patient could take to reduce the risk of firearm injury or death to themselves, their family members, or anyone who visits their home*.

1. *Communicate*
  - *Evaluate Risk: Evaluate the current circumstances to determine if having firearms in the home is right for everyone living there or visiting. Even visitors, like grandchildren, are at increased risk of firearm injury or death when they’re in a home with a firearm, particularly if the firearm is not safely stored.*
  - *Discuss Safe Storage: Ensure that your patient understands reasons for safe storage and handling practices for all firearms. In some states, you may want to remind them that they are legally responsible for keeping their firearms stored safely.*
  - *Discuss Alternative Firearm Storage: Discuss options for safe firearm storage outside of the patient’s home, such as gun ranges or retailers, especially when there is imminent risk.*
  - *Talk About Disposal of Firearms: You should talk about how to safely dispose of any unwanted firearms.*
  - *Suggest Firearm Safety Training: You can recommend that the patient take a course or attend training on firearm safety.*
  - *Discuss Other Risk Factors: As needed, explore how to reduce other risk factors for firearm injury – for instance, reducing alcohol or substance use.*

**Part IV: Firearm regulation history (20–30 minutes)**

Discuss the following:

- Why has there been no CDC-funded research on firearm violence in over 20 years?
- Given the current situation, what further studies need to be done on this issue? What creative ways can we think of to carry these out or fund them?

*This section refers back to the pre-reading, ‘The Dickey Amendment on Federal Funding for Research on Gun Violence: A Legal Dissection’ by Allen Rostron, JD. This article outlines the passing of the 1996 Dickey Amendment, which effectively curbed the CDC’s firearm violence research by stating that data couldn’t be collected for the purposes of firearm regulation advocacy. However, it was accompanied by earmarking the exact amount of funding used to study firearm violence in the year prior ($2.6 million), for research on traumatic brain injuries instead. This led to a dramatic halt in research on firearm violence. In 2011, the Dickey Amendment was extended to apply to the NIH as well, after the publication of research outlining an association between firearm possession and assaults.*

*The article also addresses the implications of the new omnibus spending bill that, in March 2018, was passed by Congress and signed by President Trump. This spending bill clarifies that the amendment does not prohibit federal funding of research on the causes of firearm violence. However, with no associated increase in funding, it is unclear if this will actually influence firearm violence research.*

*Interestingly, the article also mentions that Congressman Dickey himself changed his viewpoints on the issue before he died.*

**Wrap Up (5 minutes)**

Please spend a few minutes sharing what you learned from this session, and any questions you have remaining about this issue.

**Appendices were reviewed and updated to include the most current, unbiased terminology available at the time of publication.*

## Appendix C. Current Case in Health Policy – Firearm Violence

**Session Type: Small Group**

**One Year Follow-Up Survey**

Survey created in Qualtrics.

**Appendices were reviewed and updated to include the most current, unbiased terminology available at the time of publication.*
